# Durability of Cardiometabolic Outcomes Among Adolescents After Sleeve Gastrectomy: First Study with 9-Year Follow-up

**DOI:** 10.1007/s11695-021-05364-3

**Published:** 2021-04-10

**Authors:** Wahiba Elhag, Walid El Ansari

**Affiliations:** 1 Department of Bariatric Surgery/Bariatric Medicine, Hamad General Hospital, 3050 Doha, Qatar; 2 Department of Surgery, Hamad General Hospital, 3050 Doha, Qatar; 3 College of Medicine, Qatar University, Doha, Qatar; 4 Schools of Health and Education, University of Skovde, Skovde, Sweden

**Keywords:** Adolescents, Bariatric surgery, Laparoscopic sleeve gastrectomy, Long-term outcomes, Hypertension, Type 2 diabetes, Prediabetes, Dyslipidemia, Liver enzymes, Uric acid

## Abstract

**Background:**

Long-term durability of weight loss and comorbidity resolution beyond 7 years after laparoscopic sleeve gastrectomy (LSG) among adolescents is completely lacking.

**Methods:**

Retrospective review of adolescents aged ≤ 18 years who underwent primary LSG at our institution between 2011 and 2015 (*N* = 146). We assessed anthropometric and cardiometabolic outcomes at 1, 3, 5, 7, and 9 years.

**Results:**

Follow-up rates were 57.53%, 82.87%, 85.24%, 83.92%, and 83.33% at the five time points. The preoperative mean body mass index (BMI) (45.60 ± 6.50 kg/m^2^) decreased at year 1 (30.04 ± 4.96 kg/m^2^, *P*=0.001) and was maintained up to 9 years (30.20 ± 3.92 kg/m^2^, *P* = 0.001). Remission rates were triglycerides, 100% (11/11) at 5 years, and 100% (1/1) at 9 years; high density lipoprotein, 89.4% (17/19) at 5 years, and 100% (3/3) at 7 years; low density lipoprotein, 71.4% (11/14) and 100% (3/3) at 5 and 7 years; total cholesterol, 70% (7/10) at 5 years, and 100% (2/2) at 9 years; uric acid, 100% (3/3) at 5 years. Remission of liver enzymes was 84.6–100% (22/26–2/2) at 5–9 years. Prediabetes remission was 87.5% (14/16 and 7/8) at 5 and 7 years and 100% (3/3) at year 9. Type 2 diabetes complete remission was 50% (3/6, 1/2) at years 5 and 7, with all cases resolved at 9 years. The only case of hypertension completely resolved.

**Conclusions:**

LSG achieved substantial weight loss and remission of cardiometabolic risk factors that were sustained on the long term. This is the first study among adolescents to assess such outcomes beyond 7 years.

**Graphical abstract:**

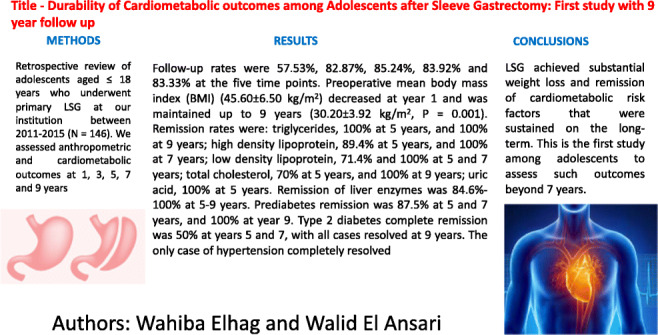

## Introduction

Severe obesity in adolescence is associated with early development of type 2 diabetes (T2DM), hypertension (HTN), dyslipidemia, nonalcoholic fatty liver disease, and cardiovascular and metabolic risk factors leading to long-term health consequences, shorter life span, and early mortality [1–3]. Lifestyle, behavioral, and pharmacological interventions result in modest short-term weight reduction but poor long-term benefits [4, 5]. Hence, bariatric surgery has recently gained attention as a treatment option for obesity in adolescents [6–8], given its good short-term benefits and promising long-term outcomes.

Generally, some data are available on the outcomes of adjustable gastric banding and Roux-en-Y gastric bypass (RYGB) among adolescents [6, 7], with most long-term studies focusing on these two procedures [6, 9–13]. Although LSG among adolescents has favorable short- and mid-term (< 5 years) results [14–18], information on its durability and long-term effects (≥ 5 years) on weight loss (WL) and comorbidity resolution are much less, despite its popularity [8, 13, 19, 20]. An exception is two studies that evaluated anthropometric parameters, diabetes remission, and body image among adolescents at 5 years after LSG [19, 20].

Therefore, the current study examined the long-term (≥ 5 years) outcomes among adolescents who underwent primary LSG. We evaluated a wide range of anthropometric and cardiometabolic variables (highlighted below). The specific objectives were to assess, preoperatively and at five time points (years 1, 3, 5, 7, and 9) after surgery, the:

- Anthropometric values at each time point compared with their preoperative values
- Cardiometabolic values at each time point compared with their preoperative values
- Long-term cardiometabolic (comorbidity) remission at the five time points

To the best of our knowledge, the current study is the first to assess the long-term (> 5 years) anthropometric or cardiometabolic outcomes among adolescents after LSG.

## Material and Method

### Study Design, Ethics, and Participants

This retrospective study was approved by the Medical Research Center (IRB) of Hamad Medical Corporation, Doha, Qatar (Protocol # 16308116). The inclusion criteria included all adolescents aged ≤ 18 years with BMI ≥ 40 or BMI ≥ 35 kg/m^2^ with comorbidities who underwent primary LSG at our Bariatric and Metabolic Surgery Centre. As the study aimed to assess long-term outcomes (i.e., follow-up for ≥ 5 years), we included all patients who had primary LSG from January 2011 to December 2015. A total of 158 adolescents underwent primary LSG during this time period. Twelve of these adolescents subsequently underwent revisional surgery and their data is presented separately. The remaining 146 patients were included in the analysis.

### Procedures and Data Collection

We searched patients’ medical charts/electronic records and retrieved pre- and post-operative data that included follow-up at 1, 3, 5, 7, and 9 years. Information included demographics [age, gender] and anthropometric [weight, height] data. We computed the BMI, BMI change, excess weight (EW), excess weight loss percentage (EWL%), WL, and total weight loss percentage (TWL%) using established formulae [21, 22]. Cardiometabolic data was also retrieved [systolic and diastolic blood pressure (SBP, DBP), triglycerides (TG), high-density lipoprotein (HDL), low-density lipoprotein (LDL), total cholesterol (TC), aspartate aminotransferase (AST), alanine aminotransferase (ALT), fasting blood glucose (FBG), hemoglobin A1c (HbA1c), uric acid].

### Definitions

In line with previous research [23], dyslipidemia was defined as having one or more of: TC ≥ 5.17 mmol/L, LDL ≥ 3.36 mmol/L, HDL ≤ 1 mmol/L, TG ≥ 1.4 mmol/L. T2DM was defined as fasting blood glucose (FBG) ≥ 7 mmol/L or HbA1c ≥ 6.5%; prediabetes as HbA1c 5.7–6.4% or FBG of 5.6–6.9 mmol/L; and hypertension was defined on the parameters outlined in international guidelines [24, 25]. Remission of dyslipidemia, T2DM, or hypertension was assessed according to the published ASMBS guidelines [26]. Uric acid remission was defined as ≤ 350 mmol/L. Weight regain was defined as regaining weight to reach BMI > 35 after successful weight loss [27]. Insufficient weight loss was defined as excess weight loss (EWL) of < 50% at 18 months after BS [28].

### Surgical Technique

Surgeries were performed by highly experienced bariatric surgeons. For primary laparoscopic sleeve gastrectomy, the procedure started with division of gastro-splenic ligament along the greater curvature 4 cm from the pylorus up to the left diaphragmatic crus with ultrasonic shears. Stomach was then mobilized and divided along the lesser curvature from antrum (4 cm from pylorus) up to the angle of His using buttressed (SeamGuard) linear 60-mm stapler (Covidien Tristapler) or Echelon Flex over the calibration tube (Midsleeve 38 Fr) introduced into the stomach. Specimen was removed through the umbilical port. Procedure was concluded with methylene blue leak test. Four adolescents (4/146, 2.7%) underwent concomitant hiatal hernia repair with the LSG.

### Bariatric and Metabolic Service

Established in 2011, the Bariatric and Metabolic Surgery Department is located in one of the largest academic tertiary care institutions in the region. The bariatric multi-disciplinary team comprises qualified bariatric surgeons and physicians, dietitians, physiotherapists, bariatric nurses, family educators, and coordinators. This institutional program evolved over the years reflected by the steady increase in bariatric surgery procedures among adolescents from 2011 to date.

In order to qualify for the Adolescent Bariatric Surgery Program in our institution, patients must have a BMI ≥ 40 kg/m^2^ or ≥ 35 with obesity-related comorbidities (e.g., asthma, diabetes, dyslipidemia, hypertension, obstructive sleep apnea, polycystic ovarian syndrome, severe nonalcoholic steatohepatitis, or substantially impaired quality of life or activities of daily living) [8]. Adolescents are referred by their pediatrician or family practice physician. Selecting a bariatric procedure is based on individualized goals of therapy (e.g., weight loss target and/or improvements in specific obesity-associated comorbidities, patient and family preferences, and personalized risk stratification that prioritizes safety).

The treatment plan from the first surgical consultation comprises multi-disciplinary assessment in the presence of family member/caretakers at each visit and includes comprehensive medical evaluation by pediatrician/bariatric physician. Adolescents also undergo a range of evaluations (as clinically indicated) including endocrinology, nutrition, pulmonary/sleep medicine, cardiology, and psychiatry assessment/s. After surgery, adolescents are routinely followed at 2 weeks, at 1, 3, 6, and 12 months, and yearly thereafter. Dieticians and physical therapists individually counsel all adolescents on routine post-surgery dietary intake and physical activity in accordance with international guidelines [8, 29]. In addition, each adolescent must see their primary care provider within a month of surgery to resume their primary care thus creating a seamless service.

### Statistical Analysis

Data were presented as proportions or mean ± standard deviation (SD) or frequency and percentage as appropriate. Pair-wise *t* tests compared the means of the continuous variables of each study participant across the given time points. Differences were considered significant at 2-tailed *p* value < 0.05. Data analysis was carried out using the Statistical Package for Social Sciences version 21 (SPSS Inc., Chicago, IL).

## Results

### Preoperative Characteristics

**Fig. 1:**
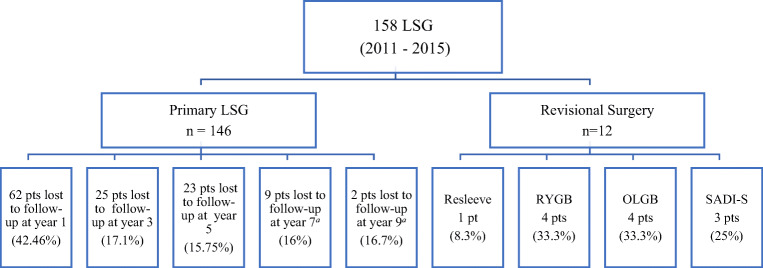
Flow diagram demonstrating loss to follow-up.*N* number, *LSG* laparoscopic sleeve gastrectomy, *pts* patients, *RYGB* Roux-en-Y gastric bypass, *OLGB* omega loop gastric bypass, *SADI-S* single anastomosis duodeno-ileal bypass with sleeve gastrectomy; loss to follow-up is not necessarily loss at all time points—a patient might skip a scheduled clinic visit, but attends the subsequent scheduled clinic visit/s, ^a^ given the study period (2011–2015) and 7–9-year follow-up; hence, the denominator at these two time points reflects the number of patients at the time of writing

The study comprised 146 adolescents, with mean age of 16.51 ± 1.29 years and nearly equal gender distribution. Mean preoperative weight and BMI were 130.40 ± 24.78 kg and 46.95 ± 7.28 kg/m^2^, respectively. The three most common comorbidities were dyslipidemia (36.3%), fatty liver (26.7%), and prediabetes (26%), while the prevalence of T2DM was 5.5% (Table 1). The follow-up rate was 57.53%, 82.87%, 85.24%, 83.92%, and 83.33% at years 1, 3, 5, 7, and 9, respectively. Figure 1 shows the diagram of loss to follow-up.

**Table 1:** Preoperative characteristics of adolescents who underwent primary LSG (*N* = 146)

Characteristic	Value
Age years (M ± SD)	16.51 ± 1.29
Gender, *n* (%)	
Male	74 (50.7)
Female	72 (49.3)
LSG surgeries per year, n*	
2011	12
2012	21
2013	23
2014	37
2015	53
Anthropometric (M ± SD)	
Weight (kg)	130.40 ± 24.78
Height (meter)	1.66 ± 0.07
BMI (kg/m^2^)	46.95 ± 7.28
EW (kg)	61.11 ± 21.48
Clinical (M ± SD)	
Systolic BP (mm/Hg)	126.43 ± 11.49
Diastolic BP (mm/Hg)	73.51 ± 8.19
Comorbidities, *n* (%)	
Dyslipidemia	53 (36.3)
Fatty liver	39 (26.7)
Prediabetes	38 (26)
Asthma	13 (8.9)
T2DM	8 (5.5)
Hypothyroidism	3 (2.1)
GERD	2 (1.4)
Seizure	2 (1.4)
HTN	1 (0.7)
Depression	1 (0.7)
OSA, PCOS, gout	0 (0)

*M ± SD* mean ± standard deviation, *BMI* body mass index, *EW* excess weight, *BP* blood pressure, *EWL%* excess weight loss percentage, *T2DM* type 2 diabetes mellitus, *HTN* hypertension, *OSA* obstructive sleep apnea, *GERD* gastroesophageal reflux disease, *PCOS* polycystic ovary syndrome

### Long-Term Anthropometric Changes

Table 2 depicts the long-term anthropometric changes at five time points. There was significant long-term weight and BMI loss throughout the study period, where the mean preoperative weight decreased from 125.97 to 82.68 kg at 9 years (*P* = 0.001), mirrored by a significant decrease in BMI (from preoperative 45.60 kg/m^2^ to 30.20 kg/m^2^, *P* = 0.001). The BMI change ranged from a mean − 16.46 ± 7.59 kg/m^2^ at 5 years to − 19.18 ± 6.52 kg/m^2^ at 9 years. Collectively, Table 2 suggests that the WL outcomes achieved at the first year after surgery were relatively maintained through the successive time points up to year 9.

**Table 2:** Long-term anthropometric changes among adolescents through five successive time points after LSG

Characteristic	Preoperative	1 year	*P*^a^	3 years	*P*^a^	5 years	*P*^a^	7 years	*P*^a^	9 years	*P*^a^
Patients *N* (%)	146 (100)	84/146 (57.53)		121/146 (82.87)		123/146 (85.24)		47/56^b^ (83.92)		10/12^b^ (83.33)	
Weight (kg)	125.97 ± 23.55	83.15 ± 16.40	*0.001*	81.36 ± 17.63	*0.001*	82.86 ± 18.70	*0.001*	84.72 ± 19.20	*0.001*	82.68 ± 6.28	*0.001*
Height (m)	1.65 ± 0.07	1.66 ± 0.07	0.384	1.66 ± 0.09	0.791	1.66 ± 0.08	0.362	1.67 ± 0.09	0.457	1.66 ± 0.11	0.765
Increase in height (m)	**—**	0.003 ± 0.03	**—**	0.002 ± 0.07	0.959	0.005 ± 0.03	0.361	0.006 ± 0.05	*0.031*	0.008 ± 0.08	0.523
BMI (kg/m^2^)	45.60 ± 6.50	30.04 ± 4.96	*0.001*	29.61 ± 6.53	*0.001*	29.80 ± 6.24	*0.001*	30.60 ± 7.58	*0.001*	30.20 ± 3.92	*0.001*
BMI change (kg/m^2^)	**—**	− 15.26 ± 6.39	**—**	− 16.41 ± 7.88	0.066	− 16.46 ± 7.59	0.448	− 18.35 ± 7.97	0.544	− 19.18 ± 6.52	0.151
EWL%	**—**	74.74 ± 23.85	**—**	81.57 ± 27.07	*0.007*	170.78 ± 137.3	*0.001*	181.32 ± 233.6	0.188	136.78 ± 38.69	0.118
WL (kg)	**—**	41.84 ± 18.50	**—**	45.69 ± 20.49	*0.007*	45.28 ± 22.18	0.050	51.31 ± 24.62	0.298	52.52 ± 21.49	0.130
TWL%	**—**	32.67 ± 11.01	**—**	35.69 ± 12.34	*0.007*	34.23 ± 12.93	0.069	36.48 ± 13.34	0.266	37.70 ± 9.83	0.133

Cell values are mean ± standard deviation, *m* meter, *BMI* body mass index, *EWL%* excess weight loss percentage, *TWL%* total weight loss percentage, *WL* weight loss, *WR* weight regain, — not applicable, italics indicate statistical significance

^a^The comparison of the value of the given year with preoperative value (pairwise *t* test); except for BMI, BMI change, EWL%, WL, and TWL% where comparison is of the given year with the 1 year value

^b^Given the study period (2011–2015) and 7–9-year follow-up, hence the denominator of these two time points reflects the number of patients at the time of writing

### Long-Term Cardiometabolic Changes

Table 3 shows the long-term cardiometabolic changes at five time points compared with preoperative values. Both the SBP and DBP showed early and durable reductions. The mean SBP decreased significantly from the preoperative 125.68 ± 0.53 to 115.16 ± 11.56 mmHg at 1 year and continued to improve significantly up to 7 years (111.18 ± 10.43 mmHg, *P* = 0.001). The DBP also significantly decreased at 1 and 3 years compared with the preoperative value (*P* = 0.042 and 0.001, respectively). At years 5, 7 and 9, the DBP was still lower than its preoperative value, but the difference was not statistically significant.

**Table 3:** Long-term cardiometabolic changes among adolescents through five successive time points after LSG

Variable	Preoperative	1 year	*P*	3 years	*P*	5 years	*P*	7 years	*P*	9 years	*P*
SBP	125.68 ± 10.53	115.16 ± 11.56	*0.001*	111.98 ± 13.25	*0.001*	115.22 ± 11.88	*0.001*	111.18 ± 10.43	*0.001*	100.0^a^	**—**
*n*	92	88		125		129		45		10	
DBP	73.40 ± 8.05	70.47 ± 8.75	*0.042*	67.56 ± 10.08	*0.001*	70.78 ± 8.41	0.074	71.56 ± 7.59	0.895	67.0^a^	**—**
*n*	92	88		125		129		45		10	
TG	1.18 ± 0.62	1.18 ± 0.62	*0.001*	0.81 ± 0.68	*0.013*	0.75 ± 0.24	*0.001*	1.31 ± 1.54	0.647	0.81 ± 0.26	0.068
*n*	131	68	52			55		15		4	
HDL	1.15 ± 0.32	1.31 ± 0.36	*0.001*	1.52 ± 0.42	*0.001*	1.53 ± 0.35	*0.001*	1.54 ± 0.25	*0.001*	1.57 ± 0.32	*0.043*
*n*	134	67		51		55		14		4	
LDL	2.91 ± 0.77	2.75 ± 0.72	*0.041*	2.34 ± 0.64	*0.001*	2.50 ± 0.78	*0.001*	2.28 ± 0.59	*0.011*	3.14 ± 0.56	0.632
*n*	133	67		51		55		14		4	
TC	4.65 ± 0.80	4.48 ± 0.76	0.056	4.19 ± 0.69	0.073	4.30 ± 0.80	*0.049*	4.30 ± 0.74	0.240	5.0 ± 0.26	0.680
*n*	135	69		52		56		14		4	
FBG	5.48 ± 2.97	4.57 ± 0.71	*0.003*	4.75 ± 1.10	*0.006*	4.82 ± 0.63	*0.028*	4.85 ± 0.66	0.156	4.45 ± 0.36	0.055
*n*	133	89		75		70		27		7	
HbA1c	6.07 ± 2.10	5.22 ± 0.51	*0.003*	5.33 ± 1.19	*0.002*	5.40 ± 0.84	*0.010*	5.25 ± 0.56	0.053	5.06 ± 0.20	0.072
*n*	120	47		45		48		16		5	
AST	21.62 ± 9.48	16.84 ± 5.59	*0.001*	16.61 ± 4.75	*0.001*	19.48 ± 7.87	0.418	19.71 ± 6.79	*0.038*	14.33 ± 0.57	0.340
*n*	123	79		74		67		22		4	
ALT	28.41 ± 18.57	15.97 ± 9.87	*0.001*	15.20 ± 7.66	*0.001*	16.43 ± 9.63	*0.001*	17.10 ± 6.22	*0.008*	7.66 ± 1.52	0.300
*n*	131	83		79		70		22		4	
Uric acid	341.37 ± 62.36	340.68 ± 74.75	0.968	281 ± 70.55	0.468	308.33 ± 41.53	0.080	**—**	**—**	**—**	**—**
*n*	65	29		21		11		5		0	

Cell values are mean ± standard deviation, *P* denotes the comparison of the value of the given year with baseline value (pairwise *t* test), *n* number of patients with data at each time point hence included in the analysis, italics indicate statistical significance

*SBP* systolic blood pressure, *DBP* diastolic blood pressure, *TC* total cholesterol, *TG* triglyceride, *HDL* high-density lipoprotein, *LDL* low-density lipoprotein, *AST* aspartate aminotransferase, *ALT* alanine aminotransferase, *FBG* fasting blood glucose, *HbA1c* hemoglobin A1c, — not applicable

Normal values: TC < 5.17, TG < 1.7, HDL > 1, LDL < 3.36, FBG 3.5–5.5, uric acid 150–350 mmol/L, HbA1c 4.8–6%, AST 13–26, and ALT 9–24 U/L

^a^No standard deviation as *n* = 1

Most lipids significantly improved across the study period compared with their preoperative values. TG decreased from a preoperative 1.18 ± 0.62 to 0.88 ± 0.33 mmol/L at year 1 and continued to significantly decrease up to year 5. Similarly, LDL significantly decreased from its preoperative value of 2.91 ± 0.77 to 2.50 ± 0.78 mmol/L and 2.28 ± 0.59 mmol/L at years 5 and 7, respectively. Moreover, HDL significantly increased at years 5, 7, and 9 compared with its preoperative value (*P* = 0.001, 0.001, 0.043, respectively). TC, however, decreased across the time points compared with its baseline value, although the difference was not statistically significant except at year 5. A point to note is that at year 9, most lipids were not significantly different from their preoperative values.

Both FBG and HbA1c significantly improved (Table 3). Compared with their preoperative levels, the mean FBG significantly decreased at years 1, 3, and 5 (*P* = 0.003, 0.006, 0.028, respectively), and HbA1c significantly improved from 6.07% to 5.22%, 5.33%, and 5.4% (*P* = 0.003, 0.002, 0.01, respectively). Likewise, AST and ALT showed long-term improvements, where both significantly decreased compared with their preoperative levels at years 1, 3, and 7 (*P* range = 0.038–0.001). Finally, uric acid exhibited a non-statistically significant reduction throughout the study compared with its preoperative value.

### Long-term Cardiometabolic Remission

There were high and sustained remission rates for all the cardiometabolic variables (Table 4). For the lipid profile, all adolescents with elevated TG had remission at 5 years and 9 years; HDL remission rate reached 89.4% at 5 years and 100% thereafter; and LDL showed durable remission (71.4% at year 5 and 100% at year 7). TC remission rate was 70% at 5 years, and all cases resolved thereafter. In terms of glycemic parameters, prediabetes remission rate increased from 50% at 5 years and 87.5% at 7 years to 100% at 9 years. Likewise, T2DM exhibited durable complete remission where 50% of adolescents had remission at year 5, and all cases resolved at 7 and 9 years after surgery. Uric acid remission was 50% at 3 years and reached 100% at 5 years.

**Table 4:** Long-term remission of cardiometabolic variables through five time points after LSG

Variable	1 year	3 years	5 years	7 years	9 years
TG	11/14 (78.5)	10/11 (90.9)	11/11 (100)	0/0 (0)	1/1 (100)
HDL	16/24 (66.6)	17/18 (94.4)	17/19 (89.4)	3/3 (100)	1/1 (100)
LDL	8/17 (47)	9/12 (75)	11/14 (71.4)	3/3 (100)	0 (0)
TC	15/21 (71.4)	9/11 (81.8)	7/10 (70)	1/1 (100)	2/2 (100)
Prediabetes	25/25 (100)	15/18 (83.3)	14/16 (87.5)	7/8 (87.5)	3/3 (100)
T2DM	4/6 (66.6)	2/5 (40)	3/6 (50)	1/2 (50)	1/1 (100)
AST	8/11 (72.7)	9 /10(90)	7/8 (87.5)	5/7 (71.4)	1/1 (100)
ALT	25/30 (83.3)	24/26 (92.3)	22/26 (84.6)	7/8 (87.5)	2/2 (100)
Uric acid	3/6 (50)	1/2 (50)	3/3(100)	**—**	**—**

Cell values are *n* (%), *TC* total cholesterol, *TG* triglyceride, *HDL* high-density lipoprotein, *LDL* low-density Lipoprotein, *AST* aspartate aminotransferase, *ALT* alanine aminotransferase, *FBG* fasting blood glucose, *HbA1c* hemoglobin A1c

Reference values: TG < 1.7 (mmol/L), HDL > 1 (mmol/L), LDL < 3.36 (mmol/L), TC < 5.17 (mmol/L), prediabetes, and T2DM based on ASMBS guidelines [26], AST13–26 (U/L), ALT 9–24 (U/L), uric acid ≤ 350 mmol/L

### Revisional Surgeries

Table 5 shows that 12 adolescents (66% females) underwent revisional surgery after a mean of 56 months. Prior to primary LSG, their mean weight was 135.12 ± 23.51 kg and BMI was 49.71 ± 5.97 kg/m^2^. After LSG, they achieved 84 ± 18.25 kg minimal weight, 30.94 ± 4.93 kg/m^2^ minimal BMI. The BMI directly before revision was 41.61 ± 4.78 kg/m^2^. Weight regain or insufficient WL were the main reasons for the revisions with exception of one adolescent who had revision because of GERD in addition to weight regain. The most common revisions were RYGB and OLGB (omega loop gastric bypass) (33.3% each) followed by SADI-S (25%), while one patient underwent resleeve.

**Table 5:** Characteristics of adolescents who underwent revisional bariatric surgery (*n* = 12)

Variable	Value
Age M ± SD	16.50 ± 1.44
Gender, *n* (%)	
Male	4 (33.3)
Female	8 (66.7)
Before primary LSG	
Anthropometric (M ± SD)	
Weight (kg)	135.12 ± 23.51
Height (meter)	1.64 ± 0.07
BMI (kg/m^2^)	49.71 ± 5.97
EW (kg)	67.40 ± 19.04
Clinical (M ± SD)	
Systolic BP (mm/Hg)	140.33 ± 3.05
Diastolic BP (mm/Hg)	76. 33 ± 19.55
Comorbidities *n* (%)	
T2DM	3 (25)
Prediabetes	3 (25)
Depression	1(8.3)
Asthma	2 (25)
Others^a^	0 (0)
After primary LSG (M ± SD)	
BMI change (kg/m^2^)	6.95 ± 4.54
EWL%	28.71 ± 17.72
Minimal weight (kg)	84 ± 18.25
Minimal BMI (kg/m^2^)	30.94 ± 4.93
Average time to revisional surgery (m)	56.41 ± 16.67
Types of revisional surgery *n* (%)	
Resleeve	1 (8.3)
RYGB	4 (33.3)
OLGB	4 (33.3)
SADI-S	3 (25)
Causes of revision *n* (%)	
Weight regain/insufficient weight loss	11 (91.7)
Weight regain + GERD	1 (8.3)
Recurrence of DM or HTN	0 (0)
Surgical complication	0 (0)
Directly before the revision	
Weight (kg)	112.17 ± 17.01
BMI (kg/m^2^)	41.61 ± 4.78

*BMI* body mass index, *EW* excess weight, *BP* blood pressure, *EWL%* excess weight loss percentage, *m* months, *RYGB* Roux-en-Y gastric bypass, *OLGB* Omega loop gastric bypass, *SADI-S* single anastomosis duodeno-ileal bypass with sleeve gastrectomy

^a^Includes hypertension, dyslipidemia, fatty liver, obstructive sleep apnea, gastroesophageal reflux disease, polycystic ovary syndrome, gout, hypothyroidism, and seizures

## Discussion

Bariatric surgery has gained ground as a strategy to reduce the adverse effects of obesity among adolescents. The findings of the present study provide important data on the longitudinal durability of LSG. Most adolescents who underwent LSG experienced substantial initial WL as well as significant remissions across several cardiometabolic risk factors. Whilst the anthropometric benefits were sustained up to 9 years, the cardiometabolic benefits were detected up to 7 years. The current study is the first to follow up a wide range of cardiometabolic outcomes of adolescents after LSG beyond 7 years.

In terms of anthropometric outcomes, the EWL% achieved by our adolescents compares favorably with findings of a systematic review among adults where the EWL% ranged between 58.4% and 62.5% at 5 to 11 years post-LSG [30]. The BMI achieved in the current sample was better than BMI reported among adolescents 5 years after LSG [31] and 8 years after RYGB (FABS-5+ study) [10]. Our superior BMI findings are possibly due to our cohort’s lower preoperative BMI compared with the FABS-5+ study. This supports the strong positive correlation between preoperative BMI and the BMI achieved in the long term [10], and also confirms that adolescents with relatively lower initial BMI accomplish more successful WL post-surgery [32]. Hence, we agree with others that operating soon after the diagnosis of obesity is established might result in better reversal of obesity and cardiometabolic risks [10].

In terms of cardiometabolic outcomes, the current study observed that both the mean SBP and DBP significantly decreased at 1 year, although in the long term, this significant reduction was maintained only for SBP (7 years). Such improvements corroborate with the Swedish Adolescent Morbid Obesity Surgery (AMOS) prospective study, where both SBP and DBP significantly decreased up to 7 years after RYGB [11]. Only one of our adolescents had hypertension, and it completely resolved at 1 year and was sustained up to 9 years (data not presented). This supports similar findings of the sustainable remission of hypertension among adolescents up to 5 years after LSG [15, 33–36]. It also agrees with the remission of hypertension among adults 5 years after LSG (the SLEEVEPASS and SM-BOSS studies) [37, 38].

Across our sample, LSG was associated with initial improvements in the lipid profile that was sustained through year 7. The adolescent Teen-LABS prospective study reported a 55% dyslipidemia remission rate 3 years after LSG [17]. Our remission rates were better than those of the Longitudinal Assessment of Bariatric Surgery (LABS) Study among adults that observed remission rates of 60%, 76.9%, and 44.4% for TG, HDL, LDL five years post-LSG [39]. Improvement in lipid profile after LSG maybe related to the better lipoprotein metabolism as a result of the WL or the decreased insulin resistance and increased glucagon like peptide-1 (GLP-1) level after surgery [11, 39, 40]. Our findings highlight the specific benefits of LSG on atherogenic lipids (LDL and TG), placing LSG as a preventive strategy against long-term cardiovascular morbidity and mortality [8].

As for prediabetes, a notable finding is that all our adolescents achieved remission, in complete agreement with the Teen-LABS study where 100% of the adolescents achieved remission [17]. As for T2DM, the remission rate in the current study ranged from 50 to 100% at 5 to 9 years after surgery. This is in agreement with similar studies among adolescents [11, 12, 17]. Parallel findings have been reported among adults after LSG, where a systematic review reported 77.8% resolution or improvement of T2DM at 5 years [30]; and longer follow-up showed a 64.7% remission rate at 10 years [41]. Our high prediabetes and T2DM remission rates confirmed that early bariatric surgery among adolescents had two-fold benefits: reversal of the progression of prediabetes to T2DM and significant and sustainable antidiabetic effects. Given such improved glycemic homeostasis, LSG should be considered early in the prevention and treatment of these two conditions in adolescence, especially for those who fail medical therapy [42]. Such improvements after bariatric surgery could be due to the decrease in insulin resistance, increase in postprandial GLP-1 levels, and enhanced beta cell function [43, 44].

Equally, our sample had substantial and durable remission of elevated liver enzymes, supporting others who reported 100% and 95.8% AST and ALT resolution 5 years after RYGB [11]. Normalizations of liver enzymes suggest that LSG could result in resolution of fatty liver disease. Likewise, for uric acid, the mean serum level exhibited some improvement at 5 years, although the difference was non-significant when compared with the preoperative level. This supports a study where uric acid was significantly elevated among adolescents with severe obesity, but was significantly reduced at 12 months after LSG (*p* < 0.028) [16]. Moreover, such reduction was significantly correlated with changes in body weight, BMI, DBP, and LDL, suggesting possible biological links between improved uric acid level and improved cardiovascular risk [16].

A point to note is that the anthropometric benefits we observed were sustained up to 9 years. However, the cardiometabolic benefits were sustained up to 7 years, as these variables at year 9 were not significantly different from their preoperative values, probably due to the small number of patients at year 9. This speculation is evidenced by the fact that most cardiometabolic variables had significant and sustained improvements across years 1–7, consistently detected due to the larger number of patients with data available for these time points (up to 129 patients). Our Bariatric Surgery Unit started in 2011, with few adolescents operated upon during 2011 (Table 1). In subsequent years, the numbers of patients increased substantially as reflected in the numbers followed up at years 1–7.

Revisional surgery for our 12 adolescents (7.5%) was due to weight regain or insufficient WL, where only one adolescent had revision due to gastroesophageal reflux disease combined with weight regain. Our revisional surgery rate was lower than that reported among adults 50 months after LSG [45], suggesting that weight regain post-LSG might be lower among adolescents than adults. Nevertheless, multidisciplinary care and close follow-up is crucial to prevent weight regain and ensure long-term success among adolescents.

### Limitations and Strengths

The current study has limitations. The retrospective design did not allow the assessment of other important outcomes, e.g., quality of life (general health perception, mental health, vitality, physical functioning, and social role functioning). Such data would have provided a more comprehensive assessment of the effects of LSG. Likewise, in retrospective interrogation of clinical datasets, data could sometimes inevitably be unavailable or of poor quality, and information on potential confounding factors could be absent. Larger numbers of patients with follow-up data at year 9 would have allowed better ability of the study to detect any significant differences at year 9 compared with preoperative values. For outcomes pertaining to fatty liver disease, the reduced levels of enzymes were taken as a biochemical proxy for remission of nonalcoholic fatty liver disease but no confirmatory biopsy was undertaken.

Nevertheless, the study has strengths. The current study is the first to assess the long-term (up to 9 years) anthropometric and cardiometabolic outcomes among adolescents after LSG. Contrary to others who reported the average changes observed cumulatively at a mean time *period* (5- to 8-year follow-up after RYGB [10]), we reported our changes at each of the five time *points* (short-, mid-, and long-term). Such ‘sharper’ reporting provided a much precise longitudinal profile of the dynamics, rich fluctuations and fine granularity of the changes and durability of the variables and comorbidity remission. Contrary to others [10, 11], the study provided detailed longitudinal (pre- and post-LSG and pre-revisional) descriptions of the characteristics of the adolescents who underwent revisional bariatric surgery, as well as their average time before revisional surgery. Such fine details provide clues to assist the bariatric team in the early identification of patients who might benefit from watchful follow-up or further intervention in order to optimize their care. We used pairwise *t* tests to detect differences between the preoperative levels and values at each of the given time points, a technique that accounts for all the values of each given participant (as opposed to group comparisons); nevertheless, such technique is highly influenced by any missing value for any given participant and any time point, hence potentially decreasing the pool of available patients for a given analysis.

## Conclusion

The findings of current study represent the first contribution to start an evidence base of the long-term outcomes of LSG among adolescents. LSG resulted in marked and durable weight loss and cardiovascular risk reduction, e.g., amelioration of prediabetes, T2DM, hypertension, dyslipidemia, elevated liver enzymes, and hyperuricemia. The study confirms that the initial benefits of bariatric surgery are sustainable up to 9 years for the great majority of adolescents, assisting them to transition into longer, healthier, and more productive adulthood lives.
